# Clinicopathologic and Prognostic Significance of Body Mass Index (BMI) among Breast Cancer Patients in Western China: A Retrospective Multicenter Cohort Based on Western China Clinical Cooperation Group (WCCCG)

**DOI:** 10.1155/2019/3692093

**Published:** 2019-04-18

**Authors:** Kang Wang, Yu-Tuan Wu, Xiang Zhang, Li Chen, Wen-Ming Zhu, Ai-Jie Zhang, Ke Zheng, Xue-Dong Yin, Fan Li, Ling-Quan Kong, Bin-Lin Ma, Hui Li, Jin-Ping Liu, Jun Jiang, Zhu-Yue Li, Yang Shi, Guo-Sheng Ren, Hong-Yuan Li

**Affiliations:** ^1^Department of the Endocrine and Breast Surgery, The First Affiliated Hospital of Chongqing Medical University, Chongqing Medical University, Chongqing, China; ^2^Department of Gynecologic Oncology, Fudan University Shanghai Cancer Center, Fudan University, Shanghai 200032, China; ^3^Department of Oncology, Shanghai Medical College, Fudan University, Shanghai 200032, China; ^4^Department of Breast and Neck Surgery, Affiliated Tumor Hospital of Xinjiang Medical University, Urumqi, Xinjiang, China; ^5^Department of Breast Surgery, Sichuan Province Tumor Hospital, Chengdu, Sichuan, China; ^6^Department of Breast Surgery, Sichuan Academy of Medical Sciences & Sichuan Provincial People's Hospital, Chengdu, Sichuan, China; ^7^Breast Disease Center, Southwest Hospital, Third Military Medical University, No. 29 Gaotanyan Street, Chongqing 400038, China; ^8^West China Hospital/West China School of Nursing, Sichuan University, China; ^9^Institute of Hospital Management, West China Hospital, Sichuan University, China; ^10^Division of Biostatistics and Data Science, Department of Population Health Sciences, Medical College of Georgia, Augusta University, Augusta, GA 30912, USA; ^11^Chongqing Key Laboratory of Molecular Oncology and Epigenetics, The First Affiliated Hospital of Chongqing Medical University, Chongqing, China

## Abstract

**Introduction:**

Clinicopathologic and prognostic significance of body mass index (BMI) in breast cancer (BC) patients remained conflicting. We aimed to investigate and modify the impact of BMI on clinicopathological significance and survival in western Chinese BC patients.

**Materials and Methods:**

8,394 female BC patients from Western China Clinical Cooperation Group (WCCCG) between 2005 and 2015 were identified. Multivariable logistic regression and Cox proportion hazard regressions were used to examine the difference of clinicopathologic and survival characteristics between BMI categories.

**Results:**

For the premenopausal, overweight and obese (OW) patients tended to have large tumor size (>5cm) (odds ratio [OR], 1.30, P<0.01) and triple-negative BC (OR, 1.31; P=0.01) compared with normal weight (NW) patients. Premenopausal underweight (UW) patients had a significantly higher risk of HER2 positive (OR, 1.71; P=0.02) and distant metastasis (OR, 2.59; P=0.01). For postmenopausal patients, OW patients showed higher risks of large tumor size (>5cm) (OR, 1.46; P=0.01), nuclear grade III (OR, 1.24; P=0.04), and lymphovascular invasion (OR, 1.46; P=0.01) compared with NW patients. An “U” shaped relationship between BMI and DFS was found (UW versus NW, adjusted hazard ratio (HR), 2.80, P<0.001; OW versus NW, adjusted HR, 1.40, P=0.02), whereas no significant difference of disease-free survival (DFS) between OW and NW premenopausal patients (adjusted HR=1.34, P=0.18) was revealed.

**Conclusion:**

We concluded that UW and OW were associated with aggressively clinicopathological characteristics, regardless of menopausal status. An “U” shaped association of BMI and DFS was revealed, and no significant difference of DFS between OW and NW in postmenopausal subgroup was revealed.

## 1. Introduction 

Breast cancer, being the most common malignant tumor in female, is rising rapidly globally and becoming one of the leading causes of cancer-related death in women [1, 2]. Meanwhile, the elevated prevalence of breast cancer contributed to obesity is becoming an important global public health burden in the past decades [3, 4]. In China, as the improvement of living standards and the lifestyles westernized, the body mass index (BMI) of the population was increased, especially among breast cancer survivors [5, 6]. Nevertheless, compared with developed countries and southeastern China, individuals in Western China were still malnutrited due to its underdeveloped economy [7]. Limited large-scale studies addressed the issues that provided evidence for modification of associations between BMI and clinicopathological or survival characteristics among breast cancer patients in Western China.

Previous findings suggested overweight and obesity are regarded as risk factors for the occurrence and development of breast cancer [3, 8–12]. Loi et al. reported that obesity was associated with larger tumors and more involved axillary nodes, but not with hormone receptor status [7]. Paradoxically, Sahin et al. found that overweight and obesity premenopausal patients had significantly less estrogen receptor- (ER-) positive tumors but more triple-negative tumors and higher stages of disease compared to normal-weighted patients, and human epidermal growth factor receptor-2 (HER-2)/luminal-like subtype was found to be significantly greater in postmenopausal overweight patients [9]. A secondary analysis of the Women's Health Initiative (WHI) randomized clinical trials reported that BMI is associated with a dose-response increased postmenopausal breast cancer risk, particularly for ER and PR positive disease [10].

It had been reported that obesity was associated with significantly more recurrence, but no significant difference in death was revealed [9]. Berclaz et al. reported that high BMI significantly influenced overall survival (OS) but not disease-free survival (DFS) [4]. Meanwhile, Fontanella et al. showed that mean DFS and OS were significantly shorter in obesity patients compared with normal weight patients [13]. However, a study from Korean showed significantly poorer outcome in underweight patients compared with that in patients with normal weight, and underweight patients had a significantly higher risk of both distant metastasis and local recurrence of breast cancer [12]. Although some previous studies had evaluated the relationship between BMI and breast cancer occurrence and survival, whether high BMI or low BMI would compromise the prognosis of breast cancer patients remains controversial, especially for Asian patients.

Herein, the aim of this retrospective multicenter study was to investigate the impact of BMI on clinicopathological significance in western Chinese breast cancer patients, especially on those aggressive characters, and assess the DFS distinction between normal, underweight, overweight, and obese breast cancer patients.

## 2. Material and Methods

### 2.1. Study Design

The data for this study was obtained from Western China Clinical Cooperation Group (WCCCG), which included 23 breast cancer centers in nine provinces of Western China (i.e., Chongqing, Sichuan, Yunnan, Guizhou, Shanxi, Gansu, Guangxi, Ningxia, and Xinjiang). The whole database included a total of 18,600 patients with breast cancer, which was histologically confirmed. Details about WCCCG had been described previously [14, 15]. Patients with primary breast cancer between January 2005 and December 2015 were potentially enrolled. We excluded patients with unknown BMI or menstrual status. ER and PR positivity were determined by immunohistochemistry when the staining of ≥1% of tumor cells appeared. Tumors were identified as HER-2-negative if they received an IHC score of 1+ and as HER2-positive only if they received an IHC score of 3+ or exhibited a HER2 gene expression level that was at least twofold higher than normal, determined by fluorescence in situ hybridization (FISH). Considering the patients in the previous period did not routinely receive immunohistochemistry for some prognostic biomarkers like Ki67, we cannot extract this variable from medical records. Despite that, the tumors were still categorized into four breast cancer subtypes according to 2013 St Gallen International Expert Consensus [16]: luminal-like, HER2/luminal-like, HER2-likel and triple-negative breast cancer. Patient medical records and WCCCG were reviewed for data regarding age at diagnosis, marital status, age at menarche, number of pregnancies and births, and clinicopathologic information.

We extracted the aforementioned patients with completed survival data including survival status and survival time, who were followed up from 2005 to 2015, and questionnaire results were obtained through phone and the outpatient department follow-up ways. Patients in every registry would answer the questions through telephone follow-up or reexamine in outpatient department at least once every three months during the first three years and then every six months thereafter. Clinical doctors would take detailed history or have a completed physical examination at each follow-up visit. Residual breast ultrasound or mammogram, chest radiography, abdominal sonography, whole-body bone scan, or PET/CT was routinely performed annually or when tumor relapse was clinically suspected. DFS was defined as the date of the diagnosis to the locoregional or distant recurrence or death from any cause, whichever came first, and DFS was considered as censored status if patients were alive until date of last contact. This observational study was entirely based on data extracted from patient medical records and was approved by the ethics committee of each participating center.

### 2.2. Statistical Method

BMI was calculated by the formula of weight (kg)/height^2^ (m^2^) and then stratified into normal weight (NW; BMI, 18.5 to 24.9 kg/m2), underweight (UW; BMI, <18.5 kg/m2), and overweight and obese (OW; BMI, >25 kg/m2) in accordance with the WHO classification [17], whose three-type groups are appreciate for Asian population. We evaluated the distribution of clinicopathological variables between patients with UW, NW, and OW groups using the Pearson *χ*2 test or Fisher's exact test for categorical variables and Student's t-test for continuous variables. Multivariable logistic regression analyses adjusting for age, tumor size, axillary lymph node status, grade, ER, and HER2 status were applied to identify independent effects of BMI on aggressive clinicopathologic characters (i.e., tumor/patients with multifocality, axillary lymph node metastasis, tumor size more than 5 cm, nuclear grade III, lymphovascular invasion, HER2-positive, triple negative, P53 positive, and distant metastasis) that were treated as binary variables (P<0.05).

We conducted log-rank tests and Cox proportion hazard regressions to examine the difference between patients with UW, NW, and OW groups in DFS and calculated hazard ratios (HRs) with 95% confidence interval (CI). Multivariate analysis was performed to acquire adjusted HRs, and recognized prognostic variables were included.

All P values reported are two-sided, which less than 0.05 were considered statistically significant. All analyses were conducted using R software (version 3.4.1).

## 3. Results

### 3.1. Patient Characteristics

**Figure 1:**
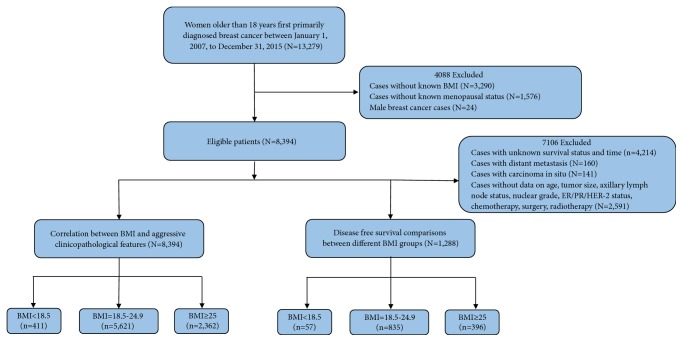
Flowchart for the data screening.

We identified 8,394 patients with invasive female breast cancer whose information on menstrual status, height, and weight was available at the initial diagnosis (Figure 1). Of the 8,394 patients, 4,462 (53.2%) were premenopausal; 3,932 (46.8%) were postmenopausal. The number of NW, UW, and OW in premenopausal group was, respectively, 3,177 (71.2%), 225 (5.0%), 1,060 (23.8%), 2,444 (62.2%), 186 (4.7%), and 1302 (33.1%) in postmenopausal group, respectively. OW patients had a higher proportion of marriage, higher frequencies of pregnancy and birth, and larger tumor and were of older age at diagnosis (all P<0.05) (Table 1). In the postmenopausal group, OW cases often tended to have higher frequencies of lymphovascular invasion, axillary lymph node metastasis, hormone receptor-positive tumors, and greater chances to receive aggressive treatments (i.e., chemotherapy, radiotherapy, and endocrine therapy), whereas we failed to observe similar tendencies in premenopausal group (Tables 2 and 3).

### 3.2. Aggressive Clinicopathological Significance of BMI in Breast Cancer Patients

For premenopausal breast cancer cases, multivariable logistic regression analyses adjusting for age, tumor size, nuclear grade, status of ER, and HER2 indicated that OW patients tended to have large tumor size (>5cm) (odds ratio [OR], 1.30; 95% CI, 1.05 to 1.62) and triple-negative breast cancer (OR, 1.31; 95% CI, 1.03 to 1.67) compared with NW patients. Meanwhile, UW patients had a significantly higher risk of HER2 positive (OR, 1.71; 95% CI, 1.02 to 2.78) and distant metastasis (OR, 2.59; 95% CI, 1.10 to 5.36) (Table 4).

Similarly, among postmenopausal patients, OW patients showed higher risks of large tumor size (>5cm) (OR, 1.46; 95% CI, 1.16 to 1.83), nuclear grade III (OR, 1.24; 95% CI, 1.00 to 1.54), and lymphovascular invasion (OR, 1.68; 95% CI, 1.04 to 2.70) when compared with NW patients. Interestingly, postmenopausal UW patients were less likely to have multifocality carcinoma (OR,0.21; 95% CI, 0.01 to 0.97) and metastasis axillary lymph nodes (OR, 0.70; 95% CI, 0.50 to 0.97) compared with NW cases (Table 4).

### 3.3. Prognostic Significance of BMI in Breast Cancer Patients

**Figure 2:**
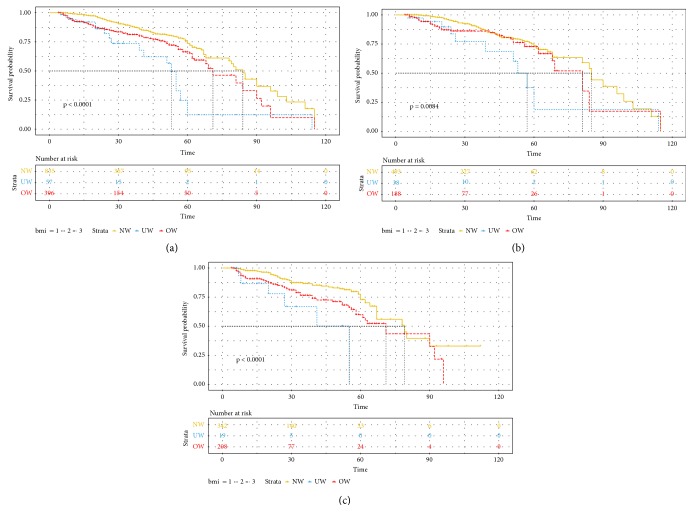
Disease-free survival comparison between UW, NW, and OW (a) whole cohort (*N* = 349), (b) premenopausal (*N* = 349), and (c) postmenopausal (*N* = 1,463) breast cancer patients. NW, normal weight (BMI, 18.5 to 24.9 kg/m2); UW, underweight (BMI, <18.5 kg/m2); OW, overweight and obese (BMI, >25 kg/m2).

We identified 1,288 patients with nonmetastatic, invasive breast cancer with complete information on clinicopathologic and survival data. Median follow-up was, respectively, 24, 20, and 23 months for NW, UW, and OW patients. 5-year DFS rates in the UW and OW group are 12.4% and 64.9%, respectively, which were significantly lower than that in the NW group (73.7%) (UW versus NW: log-rank P-value <0.001; HR=3.24, 95% CI=1.95 to 5.38; OW versus NW: log-rank P-value <0.001; HR=1.29, 95% CI= 1.13 to 1.49, Figure 2(a)). In subgroup analyses (Figure 2(b)), no significantly difference of DFS between UW and OW and NW premenopausal breast cancer patients was found (UW: HR=2.50, 95% CI= 1.31 to 4.76, P=0.006; OW: HR=1.41, 95% CI= 0.94 to 2.10, P=0.093). Inconsistently, both UW and OW patients had worse DFS compared with NW patents among postmenopausal breast cancer patients (UW: HR= 4.79, 95% CI= 2.15 to 10.69, P<0.001; OW: HR=1.84, 95% CI =1.23 to 2.74, P=0.003).

Multivariable Cox proportional hazards model including prognostic variables indicated that BMI at diagnosis, age, tumor size, number of axillary node metastasis, nuclear grade, HER-2 status, surgery type, chemotherapy, and radiotherapy were independent prognostic factors. Compared with NW group, significantly worse DFS in UW group (HR=2.80, 95% CI=1.66 to 4.73; P<0.001, Table 5) and OW group (HR=1.40, 95% CI=1.05 to 1.88; P=0.02, Table 5) were reported.

Additionally, the results of subgroup analyses were exhibited in Table 6. We found that, compared with TNBC NW patients, worse DFS in TNBC OW group (HR=2.33, 95% CI=1.06 to 5.12; P=0.04, Table 6) was reported, while in luminal-like subgroup, significantly inferior DFS was found in UW (HR=4.91, 95% CI=1.82 to 13.26; P=0.002, Table 6) compared with NW group.

## 4. Discussion

This retrospective multicenter study found that both higher and lower BMI are associated with aggressive clinicopathological characteristics and poor DFS, which were mirrored in previous studies [3, 12, 18–22]. All associations were statistically significant, apart from the difference of DFS between premenopausal OW and NW breast cancer patients, which may be due to “estrogen paradox” [23, 24].

In our analyses of linkage between BMI and aggressive clinicopathologic characteristics of breast cancer, the trend was observed that OW patients tended to have aggressive carcinoma (e.g., larger tumor size, nuclear grade III, lymphovascular invasion, and triple-negative subtype), irrespective of menstrual status. Nonetheless, UW postmenopausal patients were less likely to have carcinoma with multifocality and ALN metastasis, but UW premenopausal patients were more proven to distant metastases and HER-2 positive. High BMI had a significant positive association with ER positive breast cancer in postmenopausal patients because of increased production of circulating estrogen from the adipose tissue [25]. Accordingly, a previous study found that high BMI was an independent factor in breast cancer patients, which decreased incidence for luminal-like subtype and an increased incidence for triple-negative subtype among premenopausal patients, but those results were not shown in postmenopausal patients [3]. Similar findings also indicated premenopausal obesity patients had significantly more triple-negative subtype and higher tumor stage than that in postmenopausal obese patients [9, 25]. These results were consistent with that of our results. Conversely, a recent study reported that no significant association was determined between breast cancer characteristics and the BMI of the patients, which may be due to the limited number of patients in the study [26].

Although a previous meta-analysis [11] identifying 82 follow-up studies suggested that obesity was associated with worse overall and breast cancer specific survival in pre- and postmenopausal breast cancer, regardless of when BMI was ascertained, our results indicated that OW at diagnosis could be an independent risk factor of DFS for breast cancer patients other than premenopausal cases. Additionally, a pooled analysis of eight prospective neoadjuvant breast cancer trials assessed the impact of BMI on pathological complete response (pCR), DFS, and OS in breast cancer patients treated with neoadjuvant chemotherapy and found that higher BMI was associated with lower pCR and worse survival [13]. A study based on nationwide database of 24,698 Korean breast cancer patients reported that underweight females had a significantly higher risk of both distant metastasis and local recurrence, suggesting UW status should be included in various treatment decision-aiding tools, especially for Asian breast cancer patients [12]. Inferior DFS among UW breast cancer patients in Western China was also revealed in this study, and this finding also further validated the results from Koreans since identical ethnic variation and similar age distribution was documented. UW people were often considered as undernutrition, leading to deficiency of cytokine reactions and the subsequent activation of the immune system. It is possible that at least compromised immune system among UW patients somewhat weakened the effects of anticancer and influenced the efficacy of systemic antitumor treatments [27, 28].

As interesting as all this is, our previous dose-response meta-analysis as well as observational studies [29–33] provided solid evidence that a reduction in breast cancer incidence with the increment of BMI was found among premenopausal women or hormone replacement therapy users. Furthermore, this study also observed premenopausal OW patients had comparable DFS with premenopausal NW cases, but better than premenopausal UW patients. These phenomena called “estrogen paradox” had been proposed before [24], and the molecular mechanisms underlying a lack of effect of obesity on breast cancer risk and prognosis are complex among women with endogenous/exogenous sex hormones. It was reported that obesity would increase concentrations of circulating estrogen since the adipose tissue is a production source of estrogen, especially in postmenopausal patients [34] and for the activity of aromatase that can in turn convert androstenedione to estrone and testosterone to estradiol is strongly stimulated by both interleukin-6 (IL-6) and tumor necrosis factor-a (TNF-a), which are usually plentiful within the adipose tissue [34, 35]. Elevated level of endogenous hormones, such as estrogen and progesterone, had mitogenic and morphogenic effects on breast epithelial cells [3, 36]. But these effects on breast tissues among premenopausal women became weaker than those of postmenopausal women due to their relatively higher baseline level. Accordingly, a recently multicenter analysis using pooled individual-level data from 758,592 premenopausal women from 19 prospective cohorts indicated increased adiposity was associated with a reduced risk of premenopausal breast cancer [37]. The interaction effects of menstrual status on the association between BMI and breast cancer characteristics should be fully evaluated in further clinical studies. Furthermore, there is another hypothesis that obesity females do less breast screening, and they are more difficult to find small lumps due to too much fatty in breast, so obesity patients tend to have more advanced disease at initial diagnosis [26, 36]. However, a study by Eichholzer et al. reported no differences in mammography screening attendance between normal weight and obese and underweight women [38].

Many recent studies were conducted to examine the relationships between obesity and breast cancer risk or survival outcomes in Chinese patient cohorts [39–43]. The Shanghai Breast Cancer Survival Study suggested that postdiagnosis BMI and waist-to-hip ratio, as indicators of overall and central obesity respectively, were associated with late all-cause mortality in U-shaped pattern among long-term breast cancer survivors [42]. Another study from Northern and Eastern China indicated that general and central obesity may play different roles in different breast cancer subtypes, supporting the hypothesis that obesity affects breast carcinogenesis via complex molecular interconnections, beyond the impact of estrogen [43]. Another cohort [44] from Shanghai showed that obesity prediagnosis and weight loss postdiagnosis were inversely associated with prognosis of triple-negative breast cancer patients, suggesting maintaining a stable weight after cancer diagnosis for TNBC patients may be considered. Compared with them, we were first to supply comprehensive relationships of BMI and breast cancer clinicopathologic and survival characteristics in Western China, and multicenter and adequate sample size made these results credible.

Some limitations of our study should be acknowledged, and our results ought to be interpreted with cautions. Firstly, some important confounding factors such as socioeconomic status, performance status, status of Ki67 and P53, nuclear grade, and anti-HER2 therapy were missing in most of enrolled patients, which may have influenced our results. Then, waist circumference of patients was not measured, which was a potential modifier for the relationship of BMI and breast cancer characteristics [45]. Additionally, although the sample size in this study was only next to that in the Korean study [12] among all Asian studies, limited numbers of patients maybe lead to decline of statistical power especially in survival analyses. We cannot entirely control the quality of primary data, and pathological diagnosis from multiple hospitals will lead to inevitable bias. Last but not least, for the associations between clinicopathologic characteristics and BMI, our cross-sectional analysis could not demonstrate that abnormal weight was the cause or consequence of these clinicopathologic characteristics.

## 5. Conclusions

This multicenter retrospective cohort study found that OW at diagnosis was associated with more aggressive carcinoma (i.e., larger tumor size, nuclear grade III, lymphovascular invasion, and triple-negative subtype) than NW, regardless of menopausal status. UW premenopausal patients were more proven to distant metastases and HER-2 positive, but UW postmenopausal patients were less likely to have carcinoma with multifocality and ALN metastasis. An “U” shaped association of BMI and DFS was revealed in the whole cohort, and subgroup analyses indicated no significant difference in DFS between OW postmenopausal and NW postmenopausal cases was found. Oncologists should pay more attention to the BMI of breast cancer patients in Western China, especially for postmenopausal obese cases, and tailored treatments and surveillance should be conducted accordingly.

## Figures and Tables

**Table 1:** Demographics for eligible patients according to BMI (n=8,394).

Characteristics	BMI (kg/m2) (%)				P
	<18.5	18.5-24.9	≥25	Total	
*Menopausal status*					
Premenopausal	225(54.7)	3,177(56.5)	1060 (44.9)	4,462(53.2)	<0.001
Postmenopausal	186(45.3)	2,444(43.5)	1302(55.1)	3,932(46.8)	
*Age at diagnosis (years) *					
Mean ± SD	48.5±13.7	49.1±11.1	52.6±10.7	50.0±11.2	<0.001^a^
*Marital status*					
Married	389(94.6)	5494(97.7)	2321(98.3)	8204(97.7)	<0.001
Single/widowed/divorced	22 (5.4)	120(2.1)	35(1.5)	177(2.1)	
Missing data	0 (0)	7(0.1)	6(0.3)	13(0.2)	
*Age at menarche (years)*					
9-12	45(10.9)	693(12.3)	320(13.5)	1058(12.6)	0.02
13-15	271(65.9)	3754(66.8)	1480(62.7)	5505(65.6)	
16-18	89(21.7)	1058(18.8)	512(21.7)	1659(19.8)	
>18	6(1.5)	112(2.0)	44(1.9)	162(1.9)	
Missing data	0(0)	4(0.1)	6(0.3)	10(0.1)	
*No. of pregnancies*					
0	80(19.5)	1203(21.4)	479(20.3)	1762(21.0)	<0.001
1	100(24.3)	1523(27.1)	481(20.4)	2104(25.1)	
2	105(25.5)	1147(20.4)	522(22.1)	1774(21.1)	
3	51(12.4)	854(15.2)	357(15.1)	1262(15.0)	
4	40(9.7)	496(8.8)	248(10.5)	784(9.3)	
≥5	34(8.3)	388(6.9)	270(11.4)	692(8.2)	
Missing data	1(0.2)	10(0.2)	5(0.2)	16(0.2)	
*No. of births*					
0	82(20.0)	1114(19.8)	418(17.7)	1614(19.2)	<0.001
1	183(44.5)	2722(48.4)	876(37.1)	3781(45.0)	
2	95(23.1)	1126(20.0)	602(25.5)	1823(21.7)	
3	24(5.8)	405(7.2)	245(10.4)	674(8.0)	
4	18(4.4)	156(2.8)	136(5.8)	310(3.7)	
≥5	8(1.9)	88(1.6)	83(3.5)	179(2.1)	
Missing data	1(0.2)	10(0.2)	2(0.1)	13(0.2)	
*Laterality*					
Left	204(49.6)	2867(51.0)	1222(51.7)	4293(51.1)	0.90^b^
Right	195(47.4)	2640(47.0)	1107(46.9)	3942(47.0)	
Bilateral	4(1.0)	44(0.8)	15(0.6)	63(0.8)	
Missing data	8(1.9)	70(1.2)	18(0.8)	96(1.1)	
*Multifocality*					
No	402(97.8)	5453(97.0)	2313(97.9)	8168(97.3)	0.06
Yes	9(2.2)	168(3.0)	49(2.1)	226(2.7)	
*Initial symptoms and signs*					
Breast lump	349(84.9)	4788(85.2)	1962(83.1)	7099(84.6)	0.002^b^
Breast pain	24(5.8)	468(8.3)	215(9.1)	707(8.4)	
Nipple discharge	13(3.2)	108(1.9)	50(2.1)	171(2.0)	
Nipple inversion	4(1.0)	60(1.1)	48(2.0)	112(1.3)	
Missing data	21(5.1)	197(3.5)	87(3.7)	305(3.6)	
*Tumor size (cm)*					
≤ 1 cm	8(1.9)	136(2.4)	36(1.5)	180(2.1)	<0.001
1>, ≤ 2 cm	74(18.0)	879(15.6)	264(11.2)	1217(14.5)	
2>, ≤ 5 cm	198(48.2)	2954(52.6)	1322(56.0)	4474(53.3)	
>5 cm	41(10.0)	585(10.4)	327(13.8)	953(11.4)	
Missing data	90(21.9)	1067(19.0)	413(17.5)	1570(18.7)	
*Distant metastasis*					
No	397(96.6)	5464(97.2)	2278(96.4)	8139(97.0)	0.51
Yes	11(2.7)	105(1.9)	44(1.9)	160(1.9)	
Missing data	3(0.7)	52(0.9)	40(1.7)	95(1.1)	

**∗**Pearson *χ*2 test, except  ^a^Student's t-test and  ^b^Fisher's exact test.

**Table 2:** Clinicopathologic characteristic for premenopausal patients according to BMI (n=4,462).

Characteristics	BMI (kg/m2) (%)				P
	<18.5	18.5-24.9	≥25	Total	
*Tumor histology*					
Carcinoma in situ	4(1.8)	108(3.4)	29(2.7)	141(3.2)	0.26
Invasive carcinoma	215(95.6)	2930(92.2)	985(92.9)	4130(92.6)	
Missing data	6(2.7)	139(4.4)	46(4.3)	191(4.3)	
*Histologic type*					
Ductal	187(83.1)	2514(79.1)	846(79.8)	3547(79.5)	0.12^a^
Lobular	5(2.2)	54(1.7)	18(1.7)	77(1.7)	
Medullary	4(1.8)	39(1.2)	24(2.3)	67(1.5)	
Other	27(12.0)	526(16.6)	159(15.0)	712(16.0)	
Missing data	2(0.9)	44(1.4)	13(1.2)	59(1.3)	
*Nuclear grade*					
I	8(3.6)	136(4.3)	27(2.5)	171(3.8)	0.09
II	76(33.8)	1092(34.3)	380(35.8)	1548(34.7)	
III	31(13.8)	478(15.0)	179(16.9)	688(15.4)	
Missing data	110(48.9)	1471(46.3)	474(44.7)	2055(46.1)	
*Lymphovascular-invasion*					
No	116(51.6)	1606(50.6)	515(48.6)	2237(50.1)	0.66
Yes	4(1.8)	88(2.8)	27(2.5)	119(2.7)	
Missing data	105(46.7)	1483(46.7)	518(48.9)	2106(47.2)	
*No. of positive ALN*					
0	100(44.4)	1316(41.4)	422(39.8)	1838(41.2)	0.61
1-3	37(16.4)	558(17.6)	164(15.5)	759(17.0)	
≥4	77(34.2)	1086(34.2)	373(35.2)	1536(34.4)	
Missing data	11(4.9)	217(6.8)	101(9.5)	329(7.4)	
*ER*					
Negative	69(30.7)	851(26.8)	308(29.1)	1228(27.5)	0.25
Positive	117(52.0)	1702(53.6)	547(51.6)	2366(53.0)	
Missing data	39(17.3)	624(19.6)	205(19.3)	868(19.5)	
*PR*					
Negative	70(31.1)	891(28.0)	329(31.0)	1290(28.9)	0.13
Positive	112(49.8)	1655(52.1)	523(49.3)	2290(51.3)	
Missing data	43(19.1)	631(19.9)	208(19.6)	882(19.8)	
*HER2 *					
Negative	76(33.8)	1273(40.1)	453(42.7)	1802(40.4)	0.25
Positive	25(11.1)	305(9.6)	98(9.2)	428(9.6)	
Missing data	124(55.1)	1599(50.3)	509(48.0)	2232(50.0)	
*Tumor Subtypes*					
Luminal-like	57(25.3)	937(29.5)	311(29.3)	1305(29.2)	0.13
HER2/luminal-like	17(7.6)	187(5.9)	52(4.9)	256(5.7)	
HER2-like	8(3.6)	116(3.7)	45(4.2)	169(3.8)	
Triple negative	19(8.4)	328(10.3)	139(13.1)	486(10.9)	
Missing data	124(55.1)	1609(50.6)	513(48.4)	2246(50.3)	
*Surgery*					
Non-surgery	1(0.4)	9(0.3)	7(0.7)	17(0.4)	**<0.001** ^a^
MRM	167(74.2)	2374(74.7)	853(80.5)	3394(76.1)	
BCS	31(13.8)	391(12.3)	106(10.0)	528(11.8)	
Other	21(9.3)	301(9.5)	63(5.9)	385(8.6)	
Missing data	5(2.2)	102(3.2)	31(2.9)	138(3.1)	
*Chemotherapy*					
No	20(8.9)	326(10.3)	91(8.6)	437(9.8)	0.24
Yes	202(89.8)	2760(86.9)	940(88.7)	3902(87.4)	
Missing data	3(1.3)	91(2,9)	29(2.7)	123(2.8)	
*Radiotherapy*					
No	175(77.8)	2400(75.5)	795(75.0)	3370(75.5)	0.80
Yes	45(20.0)	673(21.2)	230(21.7)	948(21.1)	
Missing data	5(2.2)	104(3.3)	35(3.3)	144(3.2)	
*Endocrine therapy*					
No	163(72.4)	2236(70.4)	749(70.7)	3148(70.6)	0.92
Yes	57(25.3)	832(26.2)	276(26.0)	1165(26.1)	
Missing data	5(2.2)	109(3.4)	35(3.3)	149(3.3)	

ER, estrogen-receptor; PR, progesterone receptor; ALN, axillary lymph nodes; MRM, modified radical mastectomy; BCS, breast-conserving surgery.

**∗**Pearson *χ*2 test, except  ^a^Fisher's exact test.

**Table 3:** Clinicopathologic characteristic for postmenopausal patients according to BMI (n=3,932).

Characteristics	BMI (kg/m2) (%)				P
	<18.5	18.5-24.9	≥25	Total	
*Tumor histology*					
Carcinoma in situ	10(5.4)	95(3.9)	53(4.1)	158(4.0)	0.59
Invasive carcinoma	165(88.7)	2215(90.6)	1163(89.3)	3543(90.1)	
Missing data	11(5.9)	134(5.5)	86(6.6)	231(5.9)	
*Histologic type*					
Ductal	145(78.0)	1912(78.2)	997(76.6)	3054(77.7)	0.62^a^
Lobular	4(2.2)	50(2.0)	24(1.8)	78(2.0)	
Medullary	1(0.5)	16(0.7)	16(1.2)	33(0.8)	
Other	31(16.7)	410(16.8)	230(17.7)	671(17.1)	
Missing data	5(2.7)	56(2.3)	35(2.7)	96(2.4)	
*Nuclear grade*					
I	9(4.8)	128(5.2)	58(4.5)	195(5.0)	0.52
II	71(38.2)	1015(41.5)	511(39.2)	1597(40.6)	
III	30(16.1)	359(14.7)	210(16.1)	599(15.2)	
Missing data	76(40.9)	942(38.5)	523(40.2)	1541(39.2)	
*Lymphovascular-invasion*					
No	127(68.3)	1495(61.2)	693(53.2)	2315(58.9)	**0.02** ^a^
Yes	1(0.5)	43(1.8)	34(2.6)	78(2.0)	
Missing data	58(31.2)	906(37.1)	575(44.2)	1539(39.1)	
*No. of positive ALN*					
0	107(57.5)	1184(48.4)	559(42.9)	1850(47.0)	**<0.001**
1-3	26(14.0)	417(17.1)	191(14.7)	634(16.1)	
≥4	44(23.7)	703(28.8)	436(33.5)	1183(30.1)	
Missing data	9(4.8)	140(5.7)	116(8.9)	265(6.7)	
*ER*					
Negative	70(37.6)	818(33.5)	355(27.3)	1243(31.6)	**<0.001**
Positive	74(39.8)	1151(47.1)	679(52.2)	1904(48.4)	
Missing data	42(22.6)	475(19.4)	268(20.6)	785(20.0)	
*PR*					
Negative	83(44.6)	1041(42.6)	461(35.4)	1585(40.3)	**<0.001**
Positive	61(32.8)	910(37.2)	572(43.9)	1543(39.2)	
Missing data	42(22.6)	493(20.2)	269(20.7)	804(20.4)	
*HER2 *					
Negative	65(34.9)	932(38.1)	539(41.4)	1536(39.1)	0.37
Positive	23(12.4)	258(10.6)	135(10.4)	416(10.6)	
Missing data	98(52.7)	1254(51.3)	628(48.2)	1980(50.4)	
*Tumor Subtypes*					
Luminal-like	40(21.5)	634(25.9)	403(40.0)	1077(27.4)	**0.02**
HER2/luminal-like	7(3.8)	106(4.3)	63(4.8)	176(4.5)	
HER2-like	16(8.6)	149(6.1)	70(5.4)	235(6.0)	
Triple negative	25(13.4)	294(12.0)	134(10.3)	453(11.5)	
Missing data	98(52.7)	1261(51.6)	632(48.5)	1991(50.6)	
*Surgery*					
Non-surgery	1(0.5)	7(0.3)	11(0.8)	19(0.5)	0.25 ^a^
MRM	150(80.6)	1973(80.7)	1035(79.5)	3158(80.3)	
BCS	12(6.5)	140(5.7)	85(6.5)	237(6.0)	
Other	20(10.8)	234(9.6)	112(8.6)	366(9.3)	
Missing data	3(1.6)	90(3.7)	59(4.5)	152(3.9)	
*Chemotherapy*					
No	51(27.4)	440(18.0)	241(18.5)	732(18.6)	**0.005**
Yes	127(68.3)	1906(78.0)	1003(77.0)	3036(77.2)	
Missing data	8(4.3)	98(4.0)	58(4.5)	164(4.2)	
*Radiotherapy*					
No	162(87.1)	2035(83.3)	1021(78.4)	3218(81.8)	**<0.001**
Yes	15(8.1)	303(12.4)	218(16.7)	536(13.6)	
Missing data	9(4.8)	106(4.3)	63(4.8)	178(4.5)	
*Endocrine therapy*					
No	142(76.3)	1926(78.8)	945(72.6)	3013(76.6)	**<0.001**
Yes	35(18.8)	411(16.8)	296(22.7)	742(18.9)	
Missing data	9(4.8)	107(4.4)	61(4.7)	177(4.5)	

ER, estrogen-receptor; PR, progesterone receptor; ALN, axillary lymph nodes; MRM, modified radical mastectomy; BCS, breast-conserving surgery.

**∗**Pearson *χ*2 test, except  ^a^Fisher's exact test.

**Table 4:** Risk of more aggressive clinicopathological features in patients with breast cancer according to menopausal status.

Characteristics	BMI at diagnosis for premenopausal patients (kg/m2) *∗*			BMI at diagnosis for postmenopausal patients (kg/m2) *∗*		
	<18.5	18.5-24.9	25-29.9	<18.5	18.5-24.9	≥25
	OR (95% CI)	OR (95% CI)	OR (95% CI)	OR (95% CI)	OR (95% CI)	OR (95% CI)
Multifocality	1.14 (0.50, 2.27)	Reference	0.69 (0.41, 1.09)	**0.21 (0.01, 0.97)**	Reference	0.75 (0.45, 1.21)
						
ALN metastasis	0.95 (0.70, 1.28)	Reference	0.92 (0.79, 1.08)	**0.70 (0.50, 0.97)**	Reference	1.12 (0.96, 1.30)
						
Tumor size >5cm	0.87 (0.52, 1.41)	Reference	**1.30 (1.05, 1.62)**	1.16 (0.68, 1.90)	Reference	**1.46 (1.16, 1.83)**
						
Grade III	0.89 (0.56, 1.39)	Reference	1.11 (0.89, 1.38)	1.01 (0.61, 1.63)	Reference	**1.24 (1.00, 1.54)**
						
Lymphovascular invasion	0.59 (0.17, 1.47)	Reference	0.87 (0.55, 1.36)	0.30 (0.02, 1.43)	Reference	**1.68 (1.04, 2.70)**
						
HER2-positive	**1.71 (1.02, 2.78)**	Reference	0.85 (0.65, 1.12)	1.03 (0.57, 1.79)	Reference	1.01 (0.78, 1.32)
						
TNBC	0.79 (0.45, 1.32)	Reference	**1.31 (1.03, 1.67)**	1.30 (0.77, 2.11)	Reference	0.73 (0.57, 0.93)
						
P53 positive	0.66 (0.37, 1.19)	Reference	0.99 (0.73, 1.34)	0.82 (0.51, 1.34)	Reference	1.06 (0.83, 1.35)
						
Distant metastasis	**2.59 (1.10, 5.36)**	Reference	0.83 (0.44, 1.48)	0.53 (0.09, 1.74)	Reference	0.85 (0.52, 1.36)

*∗*Adjusted for age, tumor size, ALN, grade, ER, and HER2.

ALN, axillary lymph node; HER2, human epidermal growth factor receptor-2; TNBC, triple negative breast cancer.

**Table 5:** Multivariable Cox regression for DFS^*∗*^  of breast cancer patients.

Variable	Multivariate Cox Proportional Hazard Regression for DFS		
	HR†	95% CI	P value
*BMI at diagnosis (kg/m2)*			
*UW*	2.80	1.66 to 4.73	< 0.001
*NW*	Reference		
*OW*	1.40	1.05 to 1.88	0.02
*Age at diagnosis (years)*	0.98	0.96 to 1.00	0.02
*Tumor size (cm)*			
≤ 1 cm	Reference		
1>, ≤ 2 cm	2.63	0.35 to 19.73	0.35
2>, ≤ 5 cm	4.91	0.68 to 35.38	0.11
>5 cm	8.45	1.16 to 61.65	0.04
*No. of positive ALN*			
0	Reference		
1-3	1.27	0.84 to 1.92	0.25
≥4	1.72	1.23 to 2.42	0.002
*Nuclear grade*			
I	Reference		
II	1.24	0.59 to 2.59	0.57
III	2.23	1.04 to 4.79	0.04
*ER*			
Negative	Reference		
Positive	0.87	0.62 to 1.20	0.39
*PR*			
Negative	Reference		
Positive	0.76	0.53 to 1.09	0.130
*HER2 *			
Negative	Reference		
Positive	1.67	1.10 to 2.55	0.02
*Surgery*			
Non-surgery	Reference		
MRM	0.08	0.02 to 0.28	< 0.001
BCS	0.07	0.02 to 0.28	< 0.001
Others	0.07	0.02 to 0.26	< 0.001
*Chemotherapy*			
No	Reference		
Yes	0.56	0.36 to 0.89	0.01
*Endocrine therapy*			
No	Reference		
Yes	1.23	0.88 to 1.73	0.22
*Radiotherapy*			
No	Reference		
Yes	0.71	0.52 to 0.98	0.04

^*∗*^Disease-free survival (DFS) was defined as the time from surgery to the date of the first locoregional recurrence, first distant metastasis, or death from any cause.

†Multivariate analysis adjusted by BMI, age at diagnosis, tumor size, number of positive ALN, nuclear grade, status of ER, PR, and HER2, surgery, chemotherapy, endocrine therapy, and radiotherapy.

NW, normal weight (BMI, 18.5 to 24.9 kg/m2); UW, underweight (BMI, <18.5 kg/m2); OW, overweight and obese (BMI, >25 kg/m2); HR, hazard ratio; CI, confidence intervals; ALN, axillary lymph nodes; ER, estrogen-receptor; PR, progesterone receptor; HER2, human epidermal growth factor receptor 2; BCS, breast conserving surgery; MRM, modified radical mastectomy.

**Table 6:** Subgroup analyses of DFS^*∗*^  according to breast cancer patients with BMI categories.

Subgroup	BMI at diagnosis for patients (kg/m2)					
	<18.5		18.5-24.9		25-29.9	
	HR (95% CI) †	P	HR (95% CI)	P	HR (95% CI) †	P
*Overall*	2.80 (1.66, 4.73)	<0.001	Reference		1.40 (1.05, 1.88)	0.02
*Age (year)*						
<35	5.68 (1.38, 23.39)	0.02	Reference		3.72 (0.91, 15.27)	0.07
35-60	3.62 (1.80, 7.31)	<0.001	Reference		1.65 (1.16, 2.35)	0.01
>60	2.84 (0.33, 24.25)	0.34	Reference		1.03 (0.46, 2.33)	0.94
*Menopausal status*						
Premenopausal	1.99 (1.01, 3.95)	0.04	Reference		1.34 (0.87, 2.06)	0.18
Postmenopausal	7.03 (2.97, 16.63)	<0.001	Reference		1.63 (1.06, 2.50)	0.03
*Tumor size (cm)*						
<2	7.67 (1.66, 35.35)	0.01	Reference		2.39 (0.79, 7.24)	0.13
2-5	2.95 (1.48, 5.87)	0.002	Reference		1.46 (1.01, 2.12)	0.04
>5	3.05 (0.90, 10.42)	0.08	Reference		1.32 (0.73, 2.40)	0.36
*ALN metastasis*						
No	5.12 (2.44, 10.74)	<0.001	Reference		2.15 (1.21, 3.79)	0.01
Yes	1.60 (0.67, 3.80)	0.29	Reference		1.30 (0.91, 1.84)	0.15
*Nuclear grade*						
I/ II	4.86 (2.61, 9.05)	<0.001	Reference		1.31 (0.89, 1.92)	0.17
III	1.72 (0.62, 4.83)	0.30	Reference		1.48 (0.90, 2.42)	0.12
*Subtype*						
Luminal-like	4.91 (1.82, 13.26)	0.002	Reference		0.93 (0.52, 1.68)	0.82
HER2/luminal-like	3.34 (0.75, 14.81)	0.11	Reference		2.14 (1.23, 3.75)	0.01
HER2-like	6.86 (2.36, 19.90)	<0.001	Reference		1.25 (0.65, 2.39)	0.51
TNBC	0.83 (0.22, 3.11)	0.78	Reference		2.33 (1.06, 5.12)	0.04
*Chemotherapy*						
No	6.40 (1.35, 30.41)	0.02	Reference		1.03 (0.32, 3.28)	0.97
Yes	2.66 (1.50, 4.73)	0.001	Reference		1.48 (1.09, 2.02)	0.01

^*∗*^Disease-free survival (DFS) was defined as the time from surgery to the date of the first locoregional recurrence, first distant metastasis, or death from any cause.

† Multivariate analysis adjusted by BMI, age at diagnosis, tumor size, number of positive ALN, nuclear grade, status of ER, PR, and HER2, surgery, chemotherapy, endocrine therapy, and radiotherapy.

HR, hazard ratio; CI, confidence intervals; ALN, axillary lymph nodes.

## Data Availability

The Western China Clinical Cooperation Group (WCCCG) data used to support the findings of this study are available from the corresponding author upon request.

## Abbreviation

BMI: Body mass index
NW: Normal weight
UW: Underweight
OW: Overweight and obese
ALN: Axillary lymph nodes
ER: Estrogen receptor
PR: Progesterone receptor
HER2: Human epidermal growth factor receptor 2
DFS: Disease-free survival.

## References

[1] Siegel R. L., Miller K. D., Jemal A. (2016). Cancer statistics, 2016. *CA: A Cancer Journal for Clinicians*.

[2] Miller K. D., Siegel R. L., Lin C. C. (2016). Cancer treatment and survivorship statistics, 2016. *CA: A Cancer Journal for Clinicians*.

[3] Sahin S., Erdem G. U., Karatas F. (2017). The association between body mass index and immunohistochemical subtypes in breast cancer. *The Breast Journal*.

[4] Berclaz G., Li S., Price K. N. (2004). Body mass index as a prognostic feature in operable breast cancer: the international breast cancer study group experience. *Annals of Oncology : Official Journal of the European Society for Medical Oncology*.

[5] Caan B. J. (2012). Weight change and survival after breast cancer in the after breast cancer pooling project. *Cancer Epidemiology, Biomarkers & Prevention*.

[6] Wu Y., Luo Q., Li X. (2018). Clinical study on the prevalence and comparative analysis of metabolic syndrome and its components among Chinese breast cancer women and control population. *Journal of Cancer*.

[7] Dang S., Yan H., Wang D. (2014). Implication of world health organization growth standards on estimation of malnutrition in young chinese children: two examples from rural western china and the tibet region. *Journal of Child Health Care : for Professionals Working with Children in The Hospital And Community*.

[8] Blucher C., Stadler S. C. (2017). Obesity and breast cancer: current insights on the role of fatty acids and lipid metabolism in promoting breast cancer growth and progression. *Frontiers in Endocrinology*.

[9] Loi S. (2005). Obesity and outcomes in premenopausal and postmenopausal breast cancer. *Cancer Epidemiology, Biomarkers & Prevention*.

[10] Neuhouser M. L., Aragaki A. K., Prentice R. L. (2015). Overweight, obesity, and postmenopausal invasive breast cancer risk: a secondary analysis of the women's health initiative randomized clinical trials. *JAMA Oncology*.

[11] Chan D. S. M., Vieira A. R., Aune D. (2014). Body mass index and survival in women with breast cancer-systematic literature review and meta-analysis of 82 follow-up studies. *Annals of Oncology*.

[12] Moon H. G., Han W., Noh D. Y. (2009). Underweight and breast cancer recurrence and death: a report from the Korean Breast Cancer Society. *Journal of Clinical Oncology*.

[13] Fontanella C., Lederer B., Gade S. (2015). Impact of body mass index on neoadjuvant treatment outcome: a pooled analysis of eight prospective neoadjuvant breast cancer trials. *Breast Cancer Research and Treatment*.

[14] Wang K. (2016). Comparison of clinicopathological features and treatments between young (</=40 years) and older (>40 years) female breast cancer patients in west china: a retrospective, epidemiological, multicenter, case only study. *PloS One*.

[15] Wang K., Zhang X., Zheng K. (2018). Predictors of internal mammary lymph nodes (IMLN) metastasis and disease-free survival comparison between IMLN-positive and IMLN-negative breast cancer patients: Results from Western China Clinical Cooperation Group (WCCCG) database (CONSORT). *Medicine*.

[16] Goldhirsch A. (2013). Personalizing the treatment of women with early breast cancer: highlights of the st gallen international expert consensus on the primary therapy of early breast cancer 2013. *Annals of Oncology : Official Journal of the European Society for Medical Oncology*.

[17] WHO Expert Consultation (2004). Appropriate body-mass index for Asian populations and its implications for policy and intervention strategies. *The Lancet*.

[18] Wada K., Nagata C., Tamakoshi A. (2014). Body mass index and breast cancer risk in Japan: a pooled analysis of eight population-based cohort studies. *Annals of Oncology*.

[19] Cheraghi Z., Poorolajal J., Hashem T., Esmailnasab N., Doosti Irani A. (2012). Effect of body mass index on breast cancer during premenopausal and postmenopausal periods: a meta-analysis. *PloS One*.

[20] Kawai M., Minami Y., Kuriyama S. (2010). Adiposity, adult weight change and breast cancer risk in postmenopausal Japanese women: the miyagi cohort study. *British Journal of Cancer*.

[21] Jeon Y. W., Kang S. H., Park M. H., Lim W., Cho S. H., Suh Y. J. (2015). Relationship between body mass index and the expression of hormone receptors or human epidermal growth factor receptor 2 with respect to breast cancer survival. *BMC Cancer*.

[22] Daling J. R., Malone K. E., Doody D. R., Johnson L. G., Gralow J. R., Porter P. L. (2001). Relation of body mass index to tumor markers and survival among young women with invasive ductal breast carcinoma. *Cancer*.

[23] Coelingh Bennink H. J., Verhoeven C., Dutman A. E., Thijssen J. (2017). The use of high-dose estrogens for the treatment of breast cancer. *Maturitas*.

[24] Suba Z. (2013). Circulatory estrogen level protects against breast cancer in obese women. *Recent Patents on Anti-Cancer Drug Discovery*.

[25] Pierobon M., Frankenfeld C. L. (2013). Obesity as a risk factor for triple-negative breast cancers: a systematic review and meta-analysis. *Breast Cancer Research and Treatment*.

[26] Kemal Y., Demirag G., Teker F. (2015). High body-mass index is not associated with worse clinicopathological characteristics in predominantly obese breast cancer patients. *Experimental Oncology*.

[27] Mueller M., Fusenig N. (2004). Friends or foes - bipolar effects of the tumour stroma in cancer. *Nature Reviews Cancer*.

[28] DeNardo D. G., Johansson M., Coussens L. M. (2008). Immune cells as mediators of solid tumor metastasis. *Cancer and Metastasis Reviews*.

[29] Wang K., Li F., Chen L., Lai Y.-M., Zhang X., Li H.-Y. (2017). Change in risk of breast cancer after receiving hormone replacement therapy by considering effect-modifiers: a systematic review and dose-response meta-analysis of prospective studies. *Oncotarget*.

[30] Collaborative Group on Hormonal Factors in Breast Cancer (1997). Breast cancer and hormone replacement therapy: collaborative reanalysis of data from 51 epidemiological studies of 52,* *705 women with breast cancer and 108* *411 women without breast cancer. *The Lancet*.

[31] Ritte R. (2012). Adiposity, hormone replacement therapy use and breast cancer risk by age and hormone receptor status: a large prospective cohort study. *Breast Cancer Research*.

[32] van den Brandt P. A., Spiegelman D., Yaun S.-S. (2000). Pooled analysis of prospective cohort studies on height, weight, and breast cancer risk. *American Journal of Epidemiology*.

[33] Cleary M., Maihle N. (1997). The role of body mass index in the relative risk of developing premenopausal versus postmenopausal breast cancer. *Proceedings of the Society for Experimental Biology and Medicine*.

[34] Simone V., D’Avenia M., Argentiero A. (2016). Obesity and breast cancer: molecular interconnections and potential clinical applications. *The Oncologist*.

[35] Rose D., Vona-Davis L. (2014). Biochemical and molecular mechanisms for the association between obesity, chronic inflammation, and breast cancer. *Biofactors*.

[36] Wee C. C., McCarthy E. P., Davis R. B., Phillips R. S. (2004). Obesity and breast cancer screening. *Journal of General Internal Medicine*.

[37] Premenopausal Breast Cancer Collaborative Group (2018). Association of body mass index and age with subsequent breast cancer risk in premenopausal women. *JAMA Oncol*.

[38] Eichholzer M., Richard A., Rohrmann S., Schmid S., Guth U. (2016). Overweight, obesity, and breast cancer screening: results from the 2012 swiss health survey. *European Journal of Cancer Prevention : The Official Journal of the European Cancer Prevention Organisation (ECP)*.

[39] Barba M., Vici P., Pizzuti L. (2017). Body mass index modifies the relationship between *γ*-H2AX, a DNA damage biomarker, and pathological complete response in triple-negative breast cancer. *BMC Cancer*.

[40] Song Q., Huang R., Li J. (2013). The diverse distribution of risk factors between breast cancer subtypes of ER, PR and HER2: a 10-year retrospective multi-center study in China. *PLoS ONE*.

[41] Liu C., Wang Q., Sun B. (2018). Low BMI is correlated with increased TGF-*β* and IL-10 mRNA levels in the peripheral blood of breast cancer patients. *IUBMB Life*.

[42] Zhang M., Cai H., Bao P. (2017). Body mass index, waist-to-hip ratio and late outcomes: a report from the Shanghai Breast Cancer Survival Study. *Scientific Reports*.

[43] Wang F., Liu L., Cui S. (2017). Distinct effects of body mass index and waist/hip ratio on risk of breast cancer by joint estrogen and progestogen receptor status: results from a case‐control study in northern and eastern china and implications for chemoprevention. *The Oncologist*.

[44] Bao P., Cai H., Peng P. (2016). Body mass index and weight change in relation to triple-negative breast cancer survival. *Cancer Causes & Control*.

[45] Lee K., Hwang I., Han K., Jung J., Seo M. (2018). Waist circumference and risk of breast cancer in korean women: a nationwide cohort study. *International Journal of Cancer*.

